# Magnetic Polymer Composite Particles: Design and Magnetorheology

**DOI:** 10.3390/polym13040512

**Published:** 2021-02-08

**Authors:** Qi Lu, Kisuk Choi, Jae-Do Nam, Hyoung Jin Choi

**Affiliations:** 1 Department of Polymer Science and Engineering, Inha University, Incheon 22212, Korea; 22192255@inha.edu; 2 Program of Environmental and Polymer Engineering, Inha University, Incheon 22212, Korea; 3 Department of Polymer Science and Engineering, Sungkyunkwan University, Suwon 16419, Korea; kisuk929@skku.edu (K.C.); jdnam@skku.edu (J.-D.N.)

**Keywords:** magnetic polymer composite, magnetorheological, smart fluid

## Abstract

As a family of smart functional hybrid materials, magnetic polymer composite particles have attracted considerable attention owing to their outstanding magnetism, dispersion stability, and fine biocompatibility. This review covers their magnetorheological properties, namely, flow curve, yield stress, and viscoelastic behavior, along with their synthesis. Preparation methods and characteristics of different types of magnetic composite particles are presented. Apart from the research progress in magnetic polymer composite synthesis, we also discuss prospects of this promising research field.

## 1. Introduction

Over the past few decades, the development of hybrid nanocomposite particles with potential applications in multidimensional fields has led to the observation of spectacular new phenomena. Among the many nanomaterials used in industrial, environmental, and biomedical applications, magnetic nanocomposite particles have attracted considerable attention because of their immanent magnetic properties. These composites are of potential use as anticancer materials, magnetic resonance imaging [1,2], magnetic recoverable catalysts [3], hyperthermia treatment [4,5,6], bio-separation [7], drug release agents [8,9], and magnetorheological (MR) materials. However, the inherent instability of magnetic nanoparticle dispersions in a continuous phase over a long time is an urgent problem that should be addressed for facilitating the use of magnetic nanocomposite particles in various other applications. To minimize the energy generated by their large surface-volume ratio, magnetic nanoparticles tend to form aggregates. Furthermore, since pristine magnetic metal nanoparticles are highly reactive in air and easily oxidized, they could lose their magnetic properties as time goes. Therefore, from the perspective of their applications, it is of vital importance to develop protection strategies to stabilize bare magnetic particles and prevent their degradation during or after synthesis.

The synthesis of hybrid materials encapsulated in a polymer matrix has provided new possibilities for the scientific manufacture of solid-particle-forming materials. Specifically, it is important to maintain the long-term dispersion stability of magnetic polymer nanoparticles, without agglomeration and precipitation. This is particularly important for the synthesis of magnetic polymer nanoparticles from pure metals that are highly sensitive to air, such as iron, cobalt, nickel, and their alloys. Consequently, among magnetic polymer composites, iron oxides or iron-oxide-based materials are of considerable research interest, since they are more stable and easy to prepare [10,11,12,13,14,15]. On the other hand, studies have proposed the use of inorganic materials and polymers as carriers of magnetic composite materials since these composite materials can help modify the properties of the carriers for specific applications [16,17]. The fabrication of magnetic polymer particles can be divided into three categories, which are described in the next section. Generally, encapsulating magnetic particles in a polymer can inhibit not only iron leakage but also iron leakage from oxidized magnetite, resulting in better colloidal stability, increased magnetite content, and the formation of several ideal functional groups on the surface of the particles.

Actively tunable MR fluids consist of Brownian-motion-sustained nano- or microscale soft magnetic particles [18,19,20] suspended in nonmagnetic liquids such as mineral oil, natural or mineral fatty acids, silicone oil paraffin, hydrocarbon oil, and silicone oil. Moreover, various additives, such as dispersion stabilizers and surfactants can also be added to MR fluids to prevent gravity sedimentation and enhance stable particle dispersion, increase lubricity, and tune the medium viscosity [21,22,23,24,25]. When an external magnetic field field strength (*H*) is applied, the yield stress and shear viscosity of MR fluids increase by several orders of magnitude, and the MR fluids transform from a fluid-like to a solid-like phase [26,27,28,29]. Under an external magnetic field, randomly dispersed free-moving particles, which are aligned in the external magnetic field direction, in the fluids become polarized and connect to neighboring particles to form aggregate fibril-like chain structures [30,31]. Both shear viscosity and elastic modulus of the MR fluids increase sharply, and they can be controlled by adjusting the magnetic field strength. All these characteristics are closely related to the formulation of the MR fluids such as the magnetism, shape, and size of the MR particles and the medium oil viscosity [32,33]. Similar behavior is shown by MR gels and MR elastomers when the fluid medium is replaced with gels and elastomers, respectively. Since their mechanical and rheological characteristics can be accurately controlled, they have been adopted in various engineering systems such as vehicle suspensions, haptic devices [34], dampers, power steering pumps, cancer therapeutic procedures, and drug delivery systems. This review covers recent progress in the search for smart magnetic polymer particles with improved dispersion stability and various analyses of their MR characteristics. On the other hand, the electrical analog of the MR fluid, namely, the electrorheological (ER) fluid based on electro-responsive particles, such as conducting polymers and inorganic particles dispersed in a nonconducting liquid, exhibits a similar chain formation mechanism involving the dispersed particles under an applied electric field [35].

## 2. Magnetic Polymer Materials

Magnetic particles generally refer to particles containing iron-, cobalt-, and nickel-based ferromagnetic elements, alloys, oxides, or composite structures. Among different types of magnetic materials, paramagnetic materials have a magnetization proportional to the external *H* and positive susceptibility, while antimagnetic materials also have a magnetization proportional to the external *H* but have negative susceptibility. The magnetization of ferromagnetic materials initially increases significantly with *H* and later saturates when *H* reaches a certain value. Furthermore, soft magnetic particles are an important subclass of magnetic materials because of their unique importance in energy applications such as in motors, transformers, and sensors. The term “soft” is related to the intrinsic coercivity (*H_co_*). The threshold used to characterize a material as soft is arbitrary: soft magnetic materials are defined as magnetic materials with *H_co_* ≤ 400–1000 A m^−1^ [36,37]. Generally soft magnetic particles are observed in materials with a cubic crystalline structure; examples are Fe, Ni, Co, Fe–Ni, Fe–Co, and metal oxides such as MFe_2_O_4_ (where M is a divalent metal) [38].

The selection of a polymer matrix for magnetic polymer composite particles is based on the intended use of the composite particles and the choice is either commodity plastics (e.g., polystyrene (PS), polymethyl methacrylate (PMMA)) or conducting polymers (e.g., polyaniline (PANI) and polypyrrole (PPy)).

**Figure 1:**
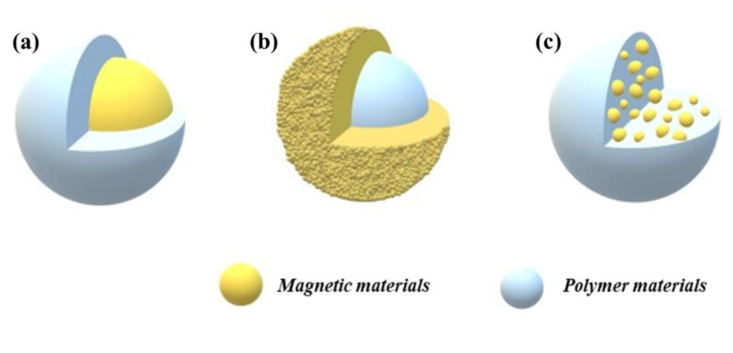
Various morphologies of magnetic polymer nanoparticles: (**a**) Magnetic core-polymer shell, (**b**) polymer core-magnetic shell, and (**c**) magnetic particles embedded in a polymeric material.

The preparation of magnetic polymer particles can be generally classified into three categories as shown in Figure 1a particles with a magnetic core-polymer shell, Figure 1b particles with a polymer core-magnetic shell, and Figure 1c magnetic particles embedded in polymeric materials. One of the strategies to prepare these structures is to synthesize magnetic nanoparticles and polymer microspheres separately and then assemble the two parts by exploiting the physical interaction between them. A second method is in situ precipitation of iron oxide nanoparticles on the polymer’s surface in the presence of polymeric microspheres. A third method is to polymerize monomers in the presence of magnetic nanoparticles; the most effective method is heterogeneous polymerization such as mini-emulsion polymerization [39], inverse emulsion/microemulsion polymerization [40,41], conventional emulsion polymerization [42], and emulsifier-free mini-emulsion polymerization [43]. These magnetic polymer composites are described in detail in the following sections.

### 2.1. Magnetic Iron-Based Polymer Composites

#### 2.1.1. Iron Oxide

Among various magnetic particles, iron oxide nanoparticles have been extensively used by the magnetic community. In particular, iron has the highest saturation magnetization at room temperature, and its Curie temperature is sufficiently high. Consequently, iron has received considerable attention in most practical applications. However, iron’s main drawback is likely to be its reactivity, especially with moisture and oxygen. Fortunately, the use of polymers as protective agents for zero-valent iron nanoparticles significantly improves their usability. Various types of iron oxides such as magnetite (Fe_3_O_4_), hematite, and gamma iron oxide (*γ*-Fe_2_O_3_) phases are available.

**Figure 2:**
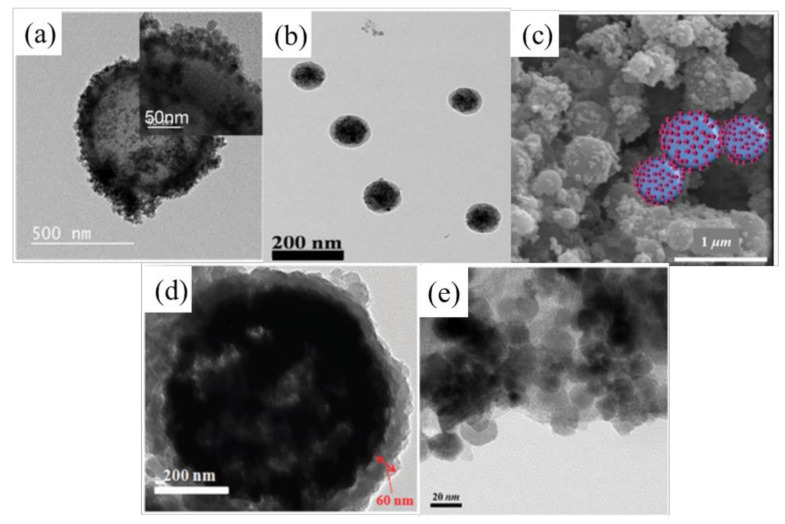
Images of iron-based magnetic polymer composites: TEM images of (**a**) hollow polydivinylbenzene@Fe_3_O_4_ (Choi et al. [44], ©American Chemical Society, 2019), (**b**) polystyrene (PS)/Fe_3_O_4_ (Kim et al. [46], ©The Polymer Society of Korea and Springer, 2018), (**d**) polypyrrole-coated Fe_3_O_4_ hybrid particles (Choi et al. [51], ©The Royal Society of Chemistry, 2013), and (**e**) PDPA/Fe_3_O_4_ composite (Dong et al. [52], © Springer Nature, 2018). (**c**) Scanning electron microscopy image of PS/Fe_3_O_4_ particles (Choi et al. [47], ©WILEY-VCH Verlag GmbH & Co. KGaA, Weinheim, Germany, 2018).

In recent years, hollow magnetic composite particles have been introduced. Because of the low particle density resulting from the cavity inside the shell of hollow particles, magnetic hollow particles can provide long-term stability, especially for MR fluid applications. Choi et al. [44] synthesized hollow polydivinylbenzene@Fe_3_O_4_ (h-PDVB@Fe_3_O_4_) nanoparticles with a relatively small density via a distillation-precipitation polymerization technique; these nanoparticles are widely used in targeted drug delivery and nano-reactor systems because of their unique structure. Uniform-sized polydivinylbenzene was first coated on silica particles, and it was followed by a SiO_2_ etching process involving hydrogen fluoride. Fe_3_O_4_ nanoparticles were then synthesized on the hollow polydivinylbenzene surface by a co-precipitation method by using FeCl_3_·6H_2_O, FeSO_4_·7H_2_O, sodium hydroxide, and so on. The more uniform the particle size, the easier the particle aggregation for a given magnetic field, which can improve the performance of the MR fluid. Figure 2a shows a transmission electron microscopy image of h-PDVB@Fe_3_O_4_ particles with a relatively narrow particle size distribution of 670 ± 33 nm. The figure shows, to some extent, that the particles can be used in MR fluids and will obtain a relatively good output. Note that in the case of an inorganic coating on the magnetic particles, Agustin-Serrrano et al. [45] reported that a silica-coated-magnetite-particle-based MR fluid showed interesting yield stress behavior at a threshold magnetic field strength.

Iron oxide composites formed with polymers such as PS or PMMA also show enhanced dispersion stability of the magnetic particles for MR applications. Kim et al. [46] fabricated Fe_3_O_4_-encapsulated PS hybrid particles via a mini-emulsion polymerization process, while Chae et al. [47] synthesized PS/Fe_3_O_4_ particles consisting of a PS surface covered with Fe_3_O_4_ nanoparticles by the Pickering emulsion polymerization technique with better dispersion stability. Note that Pickering emulsion polymerization does not require a traditional organic surface active agent, which is difficult to eliminate or recycle after polymerization, as the solid particle is used as a stabilizer. Therefore, this technique has the advantages of low toxicity, few bubbling problems, simple technology, and green environmental protection. Gao et al. [48] and Ahn et al. [49] also used Pickering emulsion polymerization to form PMMA core-Fe_3_O_4_ and Fe_2_O_3_ shell structures, respectively.

The control of the particle size of the emulsion facilitates the maintenance of the stability of the emulsion and allows new functions be provided to the emulsion. In this context, the Shirasu porous glass (SPG) membrane emulsification technique, which can provide useful water/oil/water (W/O/W) emulsion in high yield, is considered to be a mild and low-energy-consumption technique for effectively controlling the size of microcapsules. This technique improves the possibility of preparing various monodisperse polymer microspheres. Gao et al. [50] synthesized novel core/shell shaped hybrid nanoparticles of Fe_3_O_4_ embedded in PS with a narrow size distribution of 127 nm by an SPG membrane technique. Uniform homogeneous styrene monomer was first injected from the inner surface of the membrane into the outer aqueous dispersion phase by applying pressure with nitrogen. The synthesized Fe_3_O_4_ entered the styrene monomer with the help of sodium dodecyl sulfate (used as a surfactant), and it was followed by the polymerization of PS. 

Dual stimuli-response composite particles with both magnetic component and conducting polymers have aroused the interest of a wide range of researchers, including those in the fields of electrorheology and magnetorheology. Choi et al. [51] synthesized PPy-coated Fe_3_O_4_ magnetic composite particles without using an initiator and studied their double excitation response under both electric and magnetic fields. During the polymerization of PPy, Fe^3+^ in the synthesized Fe_3_O_4_, which was immersed in deionized water in advance, seeped from the surface into the polymerization reaction system, and therefore, the polymerization process could be completed without using an initiator. Dong et al. [52] synthesized organic-inorganic poly(diphenylamine) (PDPA)/Fe_3_O_4_ particles via a simple fabrication technique and examined the ER and MR characteristics of both types of particles, Kwon et al. [53] synthesized magnetic smart particles that were composites of fibrous-structured PANI and Fe_3_O_4_, and Lee et al. [54] synthesized Fe_3_O_4_/poly(*o*-anisidine) nanoparticles with a core-shell structure via oxidation polymerization. While the association of conducting polymers provided extra ER functionality, it has been found that the saturation magnetization and magnetic induction of magnetic polymer particles decreases with an increase in the polymer concentration [55]. Therefore, it is necessary to carefully modulate the size and amount of magnetic nanoparticles in these polymer magnetic composite particles to obtain the best magnetic properties [56].

#### 2.1.2. Carbonyl Iron

Soft-magnetic carbonyl iron (CI) microbeads with a high purity (>98%) and unique spherical morphology have been widely used in MR systems, and they have attracted both academia and industry because of their potential for commercial use. Nonetheless, CI particles usually have serious dispersion problems because of the significant difference in density between the dispersion medium and the magnetic particles. Furthermore, in engineering applications, bare CI particles may undergo an oxidation process with the passage of time. Many core-shell-type CI-polymer microspheres have been studied to overcome this drawback. In particular, ribbon-like PPy has been used for surface modification [57]. In this process, CI particles were initially treated with aqueous cetyltrimethylammonium bromide cationic surfactant solution and then the polymerization of PPy was initiated by adding ammonium persulfate. Although the MR effect was reduced because the CI particles were coated with polymers, the sedimentation of the liquid could remain stable for tens of hours. In another study, CI particles were coated with cholesteryl groups via a two-step reaction [58]. In this process, CI particles are first treated with (3-aminopropyl)triethoxy silane (APTES) to form amino groups on their surface and cholesteryl chloroformate is then added to CI particles coated with cholesteryl groups. This coating smoothes the surface of the magnetic material and considerably improves the thermal stability of CI under oxygen atmosphere, and the settling stability and re-dispersibility of its suspension in silicone oil. Cvek et al. [59] investigated the chemical stability and cytotoxicity of poly(glycidyl methacrylate) (PGMA)-grafted CI particles. These CI-PGMA particles, which are used in medical applications such as magnetic drug targeting systems, were found to have no cytotoxicity from an in-vitro cytotoxicity test conducted according to ISO standards. In another study, CI particles’ surface was grafted with a simple covalent coating with short poly(*n*-butyl acrylate) (PBA) chains via an atom-transfer radical polymerization (ATRP) technique [60]. First, the bare CI particles’ surface was modified with APTES and then *n*-butyl acrylate was surface-initiated on the particles. The CI-PBA particles provided a yield stress, reaching values sufficient for use in industrial applications, and showed improved chemical and sedimentation stability.

**Figure 3:**
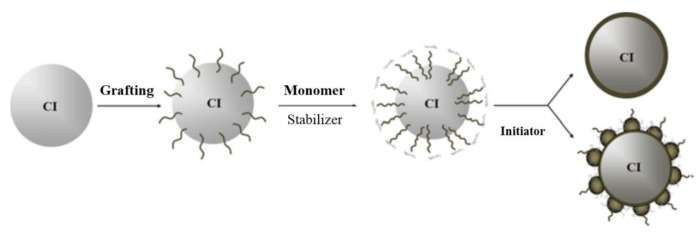
Schematic of the preparation process for polymer-coated carbonyl iron (CI) particles (Cho et al. [61], © IEEE, 2004).

Cho et al. [61] performed in situ dispersion polymerization to prepare composite particles with a CI core and a PMMA shell. The CI particle surface was grafted with organic molecules (e.g., acrylic acid or methacrylic acid) before the polymerization, as shown in Figure 3. Various conducting polymers have been used for coating CI particles, such as polyindole [62], PDPA [63], PANI [64], and polydopamine [65], through chemical oxidation polymerization to reduce the density of CI particles. These magneto-conductive polymer systems were developed not only to improve the dispersion stability of MR fluids, but also to provide great possibilities in the fields of application of both MR fluid and ER fluid systems [66].

### 2.2. Other Magnetic Meltallic Polymer Composites

**Figure 4:**
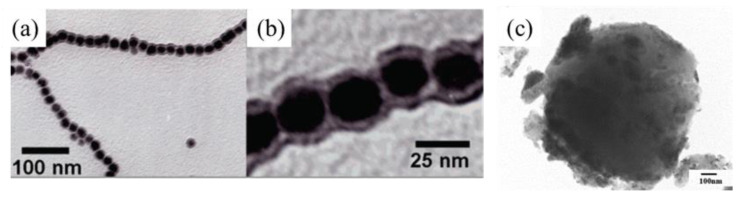
TEM images of magnetic polymer composites: (**a**,**b**) Co-PS nanoparticle chains (Korth et al. [67], ©American Chemical Society, 2006) and (**c**) Cu–Ni/polyethylene glycol magnetic polymer (Chatterjee et al. [68], Elsevier B.V., 2005).

Next to iron-based materials, cobalt-based magnetic nanoparticles are known to be the most widely used, with high magnetic susceptibility and high saturation magnetization values. However, their disadvantage is their instability, which results from their strong magnetism and van der Waals force. They tend to form agglomerates easily, and therefore, their practical application is often limited. Coating these particles with polymers is a practical and effective method for inhibiting their oxidation and agglomeration, and it improves the long-term colloidal stability of their magnetic dispersions in solution. Korth et al. [67] synthesized PS-coated cobalt nanoparticles in which the polymeric shell formed a glossy coating that encapsulated the 1D structure of the assembled nanoparticle chains. Chatterjee et al. [68] prepared copper-nickel alloy nanoparticles through chemical reduction and encapsulated them in polyethylene glycol nanoparticles by using an emulsion and a chemical cross-linking method. Spinel ferrites (M_×_Fe_3−×_O_4_), such as MgFe_2_O_4_, MnFe_2_O_4_, CoFe_2_O_4_, NiFe_2_O_4_, and ZnFe_2_O_4_ have also been combined with polymers as shown in Figure 4. Kim et al. [69] prepared raspberry-like core-shell composite particles, which comprised a PANI core and a zinc ferrite shell, through polymerization by the Pickering emulsion technique. Table 1 presents a list of magnetic polymer composites.

## 3. MR Characteristics

### 3.1. MR Fluids

**Figure 5:**
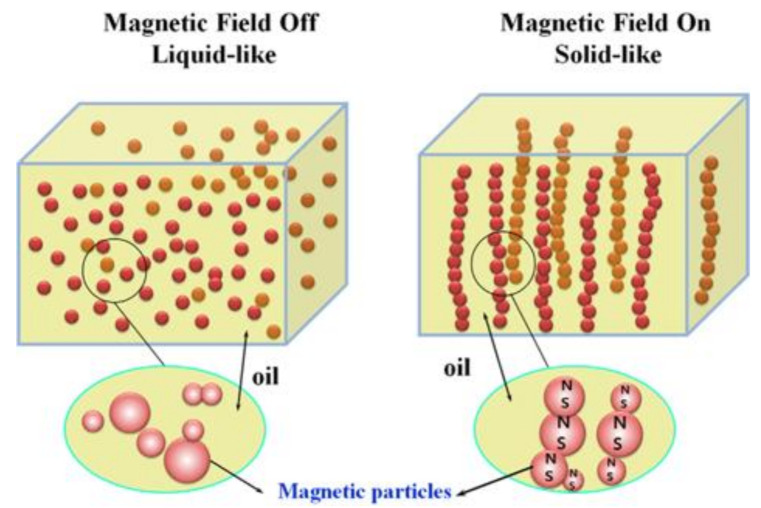
Schematic of the microstructure in a magnetorheological (MR) fluid before and after the application of an external magnetic field (Liu et al. [66], ©The Polymer Society of Korea and Springer Nature B.V., 2013.

When soft magnetic particles are suspended in nonmagnetic liquids (e.g., paraffins, natural fatty acids, minerals, hydrocarbons, and silicon-based liquids), these free-moving particles are polarized by an applied *H* and made to connect to neighboring particles aligned in the direction of the applied *H*, as shown in Figure 5 [18]. A well-known mechanism to explain MR behavior is the magnetic dipole interaction between particles because of particle magnetization. In the linear magnetization region, the magnetic moment *m* of a magnetic particle with radius *a* is field-induced and can be expressed as [51],

(1) $$m=4\pi \mu_{0}\mu_{cr}\beta a^{3}H$$

where *μ_0_* is the vacuum permeability (4*π* × 10^−7^), *μ_cr_* is the relative permeability of the medium, and *β* is the contrast factor expressed as:

(2) $$\beta =\frac{\mu_{pr}-\mu_{cr}}{\mu_{pr}-2/\mu_{cr}}$$

here, *μ_pr_* is the permeability of the particles. In this low-magnetization region, the magnetic interaction between two magnetic particles is proportional to H^2^. In a high-magnetic-field region, the magnetic moment is independent of the magnetic field strength as shown in Equation (3), where *M_s_* is the saturation magnetization of the magnetic particle. This implies that the interaction between the particles is no longer affected by an increase in the external field.

(3) $$m=4\pi \mu_{0}\mu_{cr}\beta a^{3}M_{s}$$

On the other hand, three different modes of operations have been used to describe MR suspensions: Valve (pressure) mode, shear mode, and squeeze mode. The valve mode includes stationary poles and fluids perpendicular to the direction of the magnetic field. The poles of the shear mode move parallel to the flow, and the force is in line with the direction of the magnetic field and the direction of the aggregate particle chain in the squeeze mode [70,71]. The shear mode is the most common rheological test mode used to measure the rheological properties of MR suspensions. At low shear rates, the mesoscopic structure is robust and can withstand shear-induced stress. However, hydrodynamic stress overcomes the interaction between particles and breaks the shear-induced chain mesoscopic structure, thus, starting to flow at a higher shear rate. MR suspensions exhibit a residual stress (yield stress) that increase with the magnetic field strength at that time.

**Figure 6:**
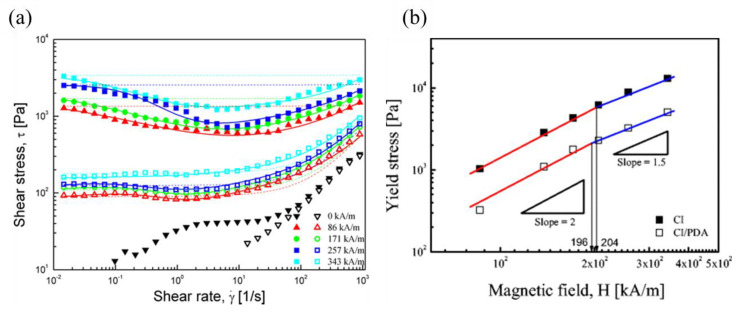
(**a**) Plots of shear stress vs. shear rate for pure Fe_2_O_3_ (closed) and PS/Fe_2_O_3_ (open) MR fluids at various magnetic field strengths (Kim et al. [74], ©American Chemical Society, 2013) with Bingham (dotted line) and Cho–Choi–Jhon (solid line) fits. (**b**) Magnetization curves of CI and CI/polydopamine powder as a function of the magnetic field strength (Kim et al. [65], ©Springer-Verlag Berlin Heidelberg, 2015).

The rheological characteristics of MR fluids can be analyzed using a simple steady shear and a dynamic mode. The flow curve of MR fluids can be obtained by performing the steady shear test, which involves two controlled shear rates and controlled shear stress methods. At a low shear rate, the mesostructure is robust and can withstand shear stress. However, as the shear rate increases, the shear stress overcomes the interaction between the magnetic particles and breaks the chain structure, and the fluid then starts to flow, defining the yield stress $\left(\tau_{y}\right)$. While the static yield stress is considered to be the minimum shear stress causing the fluid to flow, the dynamic yield stress ($\tau_{dy}$), which is the stress corresponding to the continuous failure of the aggregate, can be extrapolated using a flow curve to the zero shear rate [72]. Some rheological models for MR fluids are listed in Table 2, where *t_1_* and *t_2_* are time constants and $\eta_{\infty }$ is the viscosity at high shear rates. All of the models can predict $\tau_{dy}$ only by extrapolating the flow curve. The Bingham fluid model is the simplest model to fit the flow curve, and the Herschel–Bulkley model is also commonly used because of its simple form compared with other models. All models, except the Seo-Seo model, predict only the dynamic yield stress, which is obtained by extrapolating the shear stress curve back to zero shear rate and which is strongly influenced by the shear rate range and the selected rheological model [73,74]. However, with an increase in the shear rate, the flow is hindered more at high magnetic fields [75,76], as shown in Figure 6a. The flow shows a minimum value in the flow curve, and Kim et al. [74] used both Bingham and Cho–Choi–Jhon models to fit the flow curves of Fe_2_O_3_ and Fe_2_O_3_-PS magnetic polymer.

**Table 2:** Representative rheological models for viscoplastic yielding fluids.

Rheological Model	Equation	Ref.
Bingham	$\tau =\tau_{y}+\eta \dot{\gamma },\;\left|\tau \right|>\tau_{y}$	[77]
Herschel–Bulkley	$\tau =\tau_{y}+\eta {\dot{\gamma }}^{n},\;\left|\tau \right|>\tau_{y}$	[77]
Casson	$\sqrt{\tau }=\sqrt{\tau_{y}}+\sqrt{\eta \dot{\gamma }}$	[77]
Papanastasiou	$\tau =\tau_{y}\left(1-\exp\left(-n\dot{\gamma }\right)\right)+\eta \dot{\gamma }$	[32]
Cho–Choi–Jhon	$\tau =\frac{\tau_{y}}{1+{\left(t_{1}\dot{\gamma }\right)}^{a}}+\eta_{\infty }\left(1+\frac{1}{{\left(t_{1}\dot{\gamma }\right)}^{\beta }}\right)\;\dot{\gamma }$	[78]

Although there are many factors affecting the yield stress of MR fluids, the external magnetic field is the most dominant factor. The relationship between $\tau_{y}$ and *H* is expressed by a power law ($\tau_{y}\sim H^{n})$ [78,79]. For linear magnetic polymer particles with low permeability, *n* usually shows quadratic dependence (*n* = 2) at low magnetic fields. However, for intermediate or high field strengths, *n* is expected to be 1.5, as shown in Figure 6b [65]. A new universal yield stress correlation can be described as,

(4) $$\tau_{y}=\alpha H_{0}^{2}\left(\frac{tanh\sqrt{H_{0}/H_{c}}}{\sqrt{H_{0}/H_{c}}}\right)$$

where *α* is relevant to the susceptibility of the MR fluid and *H_c_* is the critical magnetic field strength. The parameter $\tau_{y}$ has two types of limiting behavior in relation to H:

(5a) $$\tau_{y}=\alpha H_{0}^{2}\;\;\;\;\mathrm{for}\;\;H_{0}\ll \;H_{c},$$

(5b) $$\tau_{y}=\alpha \sqrt{H_{c}}H_{0}^{3/2}\;\;\mathrm{for}\;\;H_{0}\gg \;H_{c}$$

Due to the difficulty in separating the two different regions when using Equation (4), Seo [79] proposed a rather simpler equation that could fit the experimental data well with one parameter:

(6) $$\tau_{y}\left(H_{0}\right)=\alpha H_{0}^{\frac{3}{2}}\left(1-exp\left(-m^{\prime }\sqrt{H_{0}}\right)\right)$$

Normalizing Equation (4) with *H*_c_ and setting τ_y,0_ = αH_c_^3/2^ yields the following dimensionless equation:

(7) $$\hat{\tau }={\hat{H}}^{3/2}\left(1-exp\left(-m^{\prime }\sqrt{\hat{H}}\right)\right)$$

where $\hat{H}=\frac{H}{H_{c}}$ and $m=m^{\prime }\sqrt{H_{c}}\;$.

**Figure 7:**
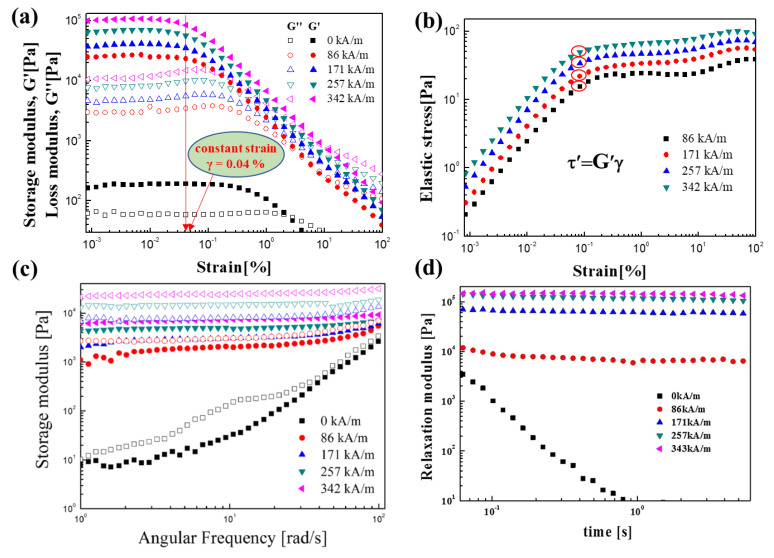
Strain amplitude sweep for an Fe_3_O_4_-PS-composite-based MR fluid (10 vol %, 100 cSt silicone oil) as a function of (**a**) storage modulus vs. strain and loss modulus vs. strain (closed symbols: $G^{\prime }$; open symbols: $G^{\prime \prime }$) and (**b**) elastic stress vs. strain (Gao et al. [48], ©Springer Nature, 2017). (**c**) Storage modulus of pure Fe_3_O_4_ (open) and PANI@Fe_3_O_4_ (closed) as a function of the angular frequency (Kwon et al. [53], ©IEEE, 2016). (**d**) Relaxation modulus of CI/PANI calculated from $G^{\prime }\left(\omega \right)$ and $G^{\prime \prime }\left(\omega \right)$ obtained from a frequency sweep test (Min et al. [64], ©Elsevier B.V., 2017).

Viscoelastic properties, including the storage $(G^{\prime }$) and loss ($G^{\prime \prime }$) modulus obtained from dynamic oscillation tests, also strongly influence the application of MR fluids. In Figure 7a, Gao et al. [50] examined $G^{\prime }$ and $G^{\prime \prime }$ as a function of strain (γ) for strain values from 0.001% to 200% at a constant frequency of 6.28 rad/s under different magnetic field strengths. In the low-strain region (linear viscoelastic range $\gamma_{LVE}$), $G^{\prime }$ and $G^{\prime \prime }$ showed a constant value, indicating that the MR fluid behaved like an elastic solid with a certain hardness. When the strain (γ) exceeded the critical value of $\gamma_{LVE}$, the $G^{\prime }$ and $G^{\prime \prime }$ curves dropped rapidly because of the deformation of the chain structure. Figure 7b shows the elastic stress, which can be defined as Equation (8). This elastic stress explains the change in the properties of the MR fluid from solid-like to liquid-like properties throughout the entire strain range, and when the solid-like structure begins to break, the yield point becomes apparent in one step by which the elastic yield can be obtained just like dynamic yield stress, which was discussed earlier:

(8) $$\tau^{\prime }=G^{\prime }\gamma$$

The angular frequency sweep test is also usually used to describe the MR properties of MR fluids. As shown in Figure 7c [53], in a previous study, the storage of Fe_3_O_4_ and PANI@Fe_3_O_4_ magnetic polymer composites was expressed as a function of the angular frequency (1–100 rad/s) for a constant γ of 0.05%. $G^{\prime }$ was considerably higher than $G^{\prime \prime }$, indicating that under the applied *H*, at $\gamma_{LVE}$, the MR fluid predominantly showed solid-like properties. Figure 7d [64] shows the relaxation modulus $G\left(t\right)$ of the CI/PANI composite as a function of time; *G*(*t*) can be determined from $G^{\prime }\left(\omega \right)$ and $G^{\prime \prime }\left(\omega \right)$ by using the Schwarzl equation [80], which is given by Equation (9) below. While the raw data of *G*^′^ and *G*^″^ is a function of ω note that ω is related to 1/t. The Schwarzl equation has been widely used not only for MR materials but also for other material systems, including polymer nanocomposites [81]:

(9) $$G\left(t\right)=G^{\prime }\left(\omega \right)-0.566G^{\prime \prime }\left(\frac{\omega }{2}\right)+0.203G^{\prime \prime }\left(\omega \right){|}_{\omega =1/t}$$

The relaxation moduli of the MR fluid saturated over time, showing distinct solid-like behavior under the applied *H* such that the strong magnetic-field-induced force between the magnetic particles did not relax with time, unlike the case without *H*.

### 3.2. MR Elastomers

**Figure 8:**
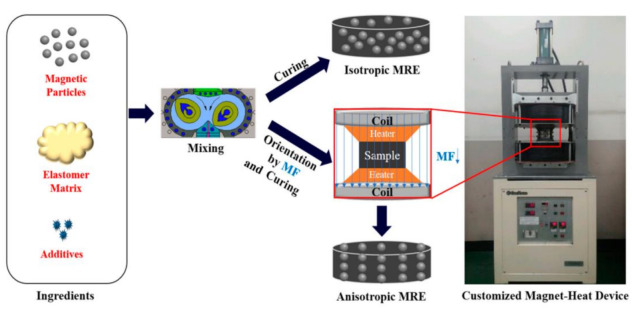
Schematic of the fabrication process for isotropic and anisotropic MR elastomers (Kwon et al. [84], ©MDPI, 2018).

MR elastomers are similar to MR fluids in terms of their physical mechanism, and they comprise magnetic particles, an elastomer matrix, and an additive. The additive plays a crucial role in the MR elastomer system in minimizing the aggregation and sedimentation of filler particles and provides lubricating properties [82]. However, compared with the fabrication method of MR fluids, there are some differences in the fabrication method used for MR elastomers as shown in Figure 8. The preparation process of MR elastomers includes mixing, curing and magnetic particle orientation. The magnetic particle chain formation is initiated in the pre-yield region, while it is activated in a post-yield continuous shear or flow regime [83]. Differently magnetized particles such as Ni [84], CoFe_2_O_4_ [85], and FeCo_3_ [86] have been studied in MR elastomers. Apart from the magnetic particles in MR elastomer systems, the MR effect of magnetic particles coated with a polymer in MR elastomers is crucial. MR elastomers have several drawbacks such as poor particle dispersibility, wettability problem, low chemical stability, and short durability [87]. These disadvantages can be eliminated by grafting CI particles with poly(trimethylsilyoxyethyl methacrylate) (PTEMATMS) via ATRP. Such grafting clearly enhances magnetostriction, the damping factor, the sensing capability, and even mechanical properties. From a MR perspective of CI-g-PTEMATMS, the $G^{\prime }$ of MR elastomers increases when CI fillers are added since rigid inorganic particles have higher stiffness property than polymer matrices [88,89]. The introduction of CI-g-PTEMATMS particles in MR elastomeric systems enhances $G^{\prime \prime }$, which can be related to the energy dissipating heat caused by friction between the matrix and particles. Furthermore, according to Fuchs et al. [90], the ATRP process has been adopted for the surface polymerization of CI particles with fluorinated styrene. The application of this process has led to remarkable durability increase. The effect of the process can be gauged from the fact that an MR elastomer with its surface coated with iron particles requires an increase of only 3% to achieve 20% strain. Whereas, an MR elastomer has to increase 17% in order to achieve the limit. MR elastomers with an iron particle coating show significant durability potential, as inferred from the force-displacement result, which can be used as a vibration isolator. In contrast to the low stiffness shown by classic elastomer composites, which is generally associated with the cross-linking of long chains, MR elastomers show higher elastic modulus, up to 30%, under the application of a magnetic field [91]. 

**Figure 9:**
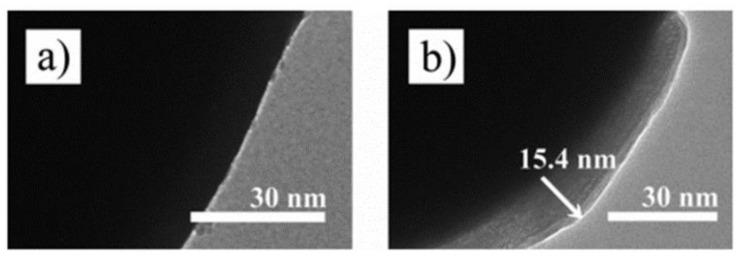
TEM images of (**a**) bare CI and (**b**) a single particle of CI-g-poly(trimethylsilyloxyethyl methacrylate (Mordina et al. [86], ©American Chemical Society, 2014).

According to Figure 9 [86], CI particles show a smooth and clean surface, whereas CI-g-poly(trimethylsilyloxyethyl methacrylate (PHEMATMS) particles have a rough surface because of the 15 nm PHEMATMS-grafted layer.

## 4. Applications of Magnetic Polymer Composite Particles

### 4.1. MR Applications

Over the past several decades, MR fluids have attracted considerable interest, and their promising properties have led to their use in many industrial applications. Parlak et al. [92] used time-dependent computational fluid dynamics analysis to simulate the motion of a piston head in an MR damper with a transient deformation mesh. A quasi-static model was proposed to calculate the damping force in the annular clearance and the specified thickness of the plug core. Yang et al. [93] developed a tiny tactile button driven by MR fluid that delivered kinesthetic information, or real button sensations, to users of small electronic devices. This button consists of a yokesleet housing containing a solenoid, plunger, elastic spring, and MR fluids. The solenoid coil is attached to the bottom of the housing surrounding the yoke, and the plunger and elastic spring are placed inside the solenoid coil. When an electric current is applied to the solenoid coil, the flux fluid is activated in the gap between the yoke and the plunger, and it forms a granular chain. The MR fluid chains produce shear stresses that are transmitted to the user in the form of resistance on the contact plate.

**Figure 10:**
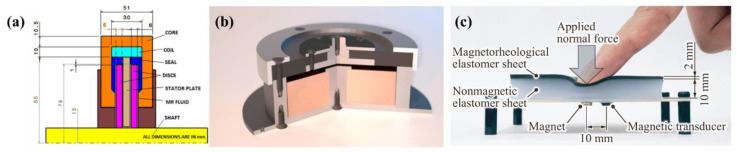
Applications of (**a**) a finite element analysis model of a proposed MR brake (Patil et al. [96], ©Elsevier Ltd., 2016), (**b**) a sectional view of an active damper based on an MR elastomer (Mikhailov et al. [103], ©Elsevier B.V., 2016), and (**c**) the basic structure of a flexible tactile sensor (Kawasetsu et al. [108], ©MDPI, 2018).

Chen et al. [94] developed an MR actuator for use as an intelligent actuator for auxiliary knee stents. It could be used to provide controllable torque in the auxiliary knee stent. It comprised a DC motor, an external cylinder connected to the upper leg, an MR fluid for the shearing mode to generate or transmit torque, and an internal cylinder connected to the shaft. Lee et al. [95] developed a prototype tactile display containing MR fluid. This tactile display device simulated the skin of the fingers to sense sensations of contact, such as compliance, friction, and surface topography. Patil et al. [96] introduced a finite element analysis model and an analysis method for analyzing an MR brake in detail as presented in Figure 10a. In another study, a model was developed to describe centrifugation, and an MR clutch design was developed to alleviate the sealing problem [97].

Choi et al. [98] proposed a semiactive controlled MR suspension for vibration control of passenger car engines. The upper part of the mount comprised the main rubber to provide appropriate stiffness and damping properties. The MR fluid moved through the gap between the shell and the magnetic pole and was controlled by the magnetic field strength. Lee et al. [99] used a biopolymer of xanthan gum (XG) to coat the surface of CI particles to improve the MR polishing performance. In existing commercial systems, the aqueous MR fluids used in QED technology polishing systems comprise CI particles, nonmagnetic abrasives, deionized water, and stabilizers. When the nonmagnetic MR fluid is moved to the gap on the wheel, the appropriate part of the gap is magnetized by an electromagnet. The magnetized MR fluid removes material and is then transported outside the magnetic field, where it is removed from the wheel. In another study a novel tactile device using MR fluid was presented. It could provide rejection information about an organ to a surgeon [100]. When the diaphragm was compressed by the operator, the operator could feel the touch of the MR fluid, and the sense of touch could be controlled through magnetic fields.

General applications of MR elastomers include adaptively tuned vibration absorbers (TVAs), vibration isolators, dampers, vehicle shock absorbers, sandwich beams, actuators, and sensors. The intelligent behavior of MR elastomers, which work in the shear mode, has been practically applied in automobile suspensions. Ginder et al. [101] developed a suspension bushing, which is a pioneer application of MR elastomers. The TVA developed by them is a single-degree-of-freedom system that can increase the natural frequency from 500 Hz to 610 Hz in the range of the maximum available magnetic field. MR elastomers are also used to develop active or semiactive isolators that can work in both horizontal and vertical directions. For example, Yang et al. [102] developed an isolator that works in the shear mode with an MR elastomer, Mikhailov et al. [103] developed an active damper that could be used for micro- or nano-positioning drives of microisolator objects as shown in Figure 10b, and Sun et al. [104] developed MR elastomer isolators to protect multistore buildings from the impact of seismic events.

Adaptive sandwich structures are another application for which MR elastomers are potential candidate materials. Dyniewicz et al. [105] studied the behavior of sandwich beams by placing MR cores at the ends of beams, and Szmidt et al. [106] proposed a new type of MR-elastomer-based sandwich beam in which the MR elastomer is clamped at the edge with two separate cantilevered beams. MR elastomers are also potential candidate materials for sensor components. Ge et al. [107] developed a displacement sensor by coating a porous MR elastomer with carbon nanotubes. A tactile sensor is another type of sensing device in the field of MR elastomers, Kawasetsu et al. [108] developed a flexible tactile sensor based on an MR elastomer that could detect normal forces and vertical deformation in an application as given in Figure 10c, and Lee et al. [109] developed a micro-cantilever beam with MR elastomer characteristics. More recently, Xu et al. [110] used an MR elastomer as a polishing composite material to perform magnetically controlled polishing.

MR elastomers are also used in biomedical applications, for purposes such as drug and cell delivery for active burns. Fahrni et al. [111] created high-aspect-ratio lying artificial cilia with a length of 300 μm based on lithographic techniques. In another study, a new type of an active porous scaffold controlled by a magnetic field was used to transfer different biological agents by alginate brine gel and magnetite particles [112]. In yet another study, the engineering adjustable active surface morphology of micron-scale columns was developed by using magnetic polymer particles controlled by an electromagnetic field to prevent serious infections from being caused by the formation of biofilms on medical devices [113].

### 4.2. Other Applications

Owing to their physical and mechanical properties, polymer-encapsulated magnetic filler particles with a diameter of less than 1 μm have been widely used in pharmaceutical, cosmetic, paint, and other industries. Magnetic separation of labeled cells and other biological entities, therapeutic drugs, gene and radionuclide delivery, radiofrequency methods of tumor catabolism through hyperthermia, and contrast enhancers for magnetic resonance imaging applications are the most important applications of magnetic polymer composite particles [114].

There are several ways to minimize environmental contaminants using magnetic nanoparticles. Iron is one of the strong agents that degrades a wide range of organic and inorganic materials in contaminated chlorinated solvents and water sources. Moreover, surface sorption and co-precipitation facilitate the removal of contaminants in air or water environments through the generation of iron oxide/hydroxide [56,115,116,117]. Hybrid magnetic nanoparticles have been developed using PMMA and super-paramagnetic iron oxide nanoparticles for the removal of heavy metals such as Cu, Co, Hg, and Pb under a magnetic field [118]. The possible contaminant removal mechanisms when magnetic nanoparticles are used include co-precipitation, electrostatic attraction, and high reductive reactivity between molecules [100]. Furthermore, magnetic nanoparticles can be used as nonabsorbents for water purification, and they can increase the removal efficiency of contaminants by acting as immobilization carriers [118].

Ligand exchange methods have been developed for prefabricated magnetic nanoparticles, to overcome the limitation of functional surfactants. Extensive studies on the functionalization of gold and semiconductor nanoparticles through ligand exchange strategies have been reported, demonstrating the feasibility of this approach at the nanoscale. Ligating groups such as thiols, COOH, diols, and phenols have been used to coordinate magnetic nanoparticles. Fauconnier et al. [119] investigated the 2,3-dimercaptosuccinic acid onto electrostatically stabilized maghemite colloids to conjugate the proteins onto thiolated magnetic nanoparticles. Rahj et al. [120] systematically studied the modification of bidentate enediol compounds containing Fe nanoparticles by using a series of compounds and evaluated them by X-ray absorption spectroscopy. On the basis of this research, Xu et al. [121] developed a bifunctional small molecule dopamine surfactant to functionalize magnetic nanoparticles through ligand exchange. In addition, dendritic coating functionalization of magnetic nanoparticles was also achieved. Through ligand exchange reaction with polyamide dendrites, Kim et al. [122] prepared robust and dimensionally stable coatings.

The functionalization of magnetic nanoparticle terminal polymer corona was studied using surface initiated polymerization, which can precisely change the molar mass and composition of the polymer chains growing on a colloidal surface. Wang et al. [123] used a combination of ligand exchange and surface-initiated ATRP to prepare core-shell PS-grafted γ-Fe_2_O_3_ nanoparticles. Marutani et al. [124] and Ninjbadgar et al. [125] demonstrated that PMMA-coated core-shell colloids could be prepared by covalently anchoring the ATRP-starting sites to magnetite nanoparticles using silane coupling agents. Gelbrich et al. [126] prepared thermo-responsive magnetic core-shell nanoparticles by using surface-initiated ATRP of 2-methoxy methyl methacrylate from a Fe_3_O_4_ colloidal initiator. Surface-initiated nitroxide-mediated polymerization has also been investigated for the preparation of polymer-coated core-shell nanoparticles. Matsuno et al. [127] synthesized a phosphoric acid functional Hawker-type alkoxyamine based on TEMPO to bind the initiator portion tightly to the iron oxide surface.

## 5. Conclusions

Functionalized magnetic polymer nanoparticles have high potential for development. These magnetic polymer nanoparticles have broad application prospects in the fields of nonmagnetic drug delivery, biomarker and biological modification, etc. [128,129,130,131]. However, a thin polymer coating cannot protect reactive magnetic particles against oxidation. Another disadvantage of polymer-coated nanomagnetic particles is that the thermal stability of their coating at elevated temperatures is relatively low. Therefore, in the final application, ensuring the careful regulation of the thickness of the polymer coating may be a solution for the synthesis of magnetic polymer composites by using appropriate methods.

In this review, magnetic polymer composite particles are introduced by their composition (including two parts: Magnetic particles of iron-based materials such as Fe_2_O_3_, Fe_3_O_4_, and CI and other magnetic particles such as cobalt and spinel ferrites, and polymers such as PS, PMMA, PEG, and PANI) and synthetic methods. Fe_3_O_4_ is the most attractive magnetic particle owing to its high saturation magnetization, soft magnetic behavior, easy synthesis, suitable particle shape and size, and low density. The most important role of a polymer coating on the surface of magnetic particles is that a less dense polymer shell can significantly improve the sedimentation stability of MRF. Furthermore, coating magnetic particles with a conductive polymer enables the composite to conduct electricity, and this material can be used in systems that provide a dual stimulus response in both electric and magnetic fields [132].

Magnetic polymer materials have also been widely used in MR fluid and MR elastomer systems. The flow curve of MR elastomers can be fitted with a variety of rheological models to find the yield stress, which is an important parameter of MR fluids. The relationship between τ_ν_ and *H* is expressed by a power law (τ_ν_~*H*^n^) in which *n* changes with the magnetic field strength from 2 to 1.5 [133]. Viscoelastic properties can be determined through dynamic oscillation tests. Of all the magnetic particles, CI-based magnetic polymers show relatively excellent MR effect, but the sedimentation stability of these composites is still a problem that should be resolved. Furthermore, polymer-coated iron particles used in MR elastomers also show significant durability potential in the force-displacement result. Finally, we have provided a brief overview of the applications and prospects of magnetic polymer composites. It is hoped that these applications will lead to fruitful results and help expand their commercial application.

## Figures and Tables

**Table 1:** Preparation methods for various magnetic polymer composites and their magnetic properties (the abbreviation “N.D.” denotes “not determined”).

Magnetic Particles	Polymer	Method	Density [g/cm^3^]	*M*_s_ Value [emu/g]	Ref.
Fe_3_O_4_	Polydivinylbenzene	Distillation-precipitation polymerization	1.83	41	[44]
Fe_3_O_4_	Poly(diphenylamine)	Coprecipitation	2.44	77.1	[52]
Fe_3_O_4_	Poly(*o*-anisidine)	Chemical oxidation polymerization	2.52	36	[54]
Fe_3_O_4_	Polystyrene	Mini-emulsion polymerization	2.8	27	[46]
Fe_3_O_4_	Polystyrene	Pickering emulsion polymerization	1.69	59	[47]
Fe_3_O_4_	Polystyrene	Shirasu porous glass membrane technique	2.29	31.7	[48]
Fe_2_O_3_	Poly(methyl methacrylate)	Pickering emulsion polymerization	1.68	20.05	[50]
Fe_3_O_4_	Polyaniline	Micelle-assisted self-assembly method	N.D.	39.4	[53]
CI	Polypyrrole	In situ polymerization	N.D.	≈120	[57]
CI	Cholesteryl groups	Surface modification	N.D.	≈180	[58]
CI	Poly(glycidyl methacrylate)	Atom transfer radical polymerization	N.D.	215	[59]
CI	Poly(*n*-butyl acrylate)	Atom transfer radical polymerization	N.D.	227	[60]
CI	Polyindole	Chemical oxidation polymerization	7.3	180	[62]
CI	Poly(diphenylamine)	Oxidative dispersion polymerization	7.42	191	[63]
CI	Polyaniline	Chemical oxidation polymerization	4.21	136	[64]
CI	Polydopamine	In situ self-oxidative polymerization	6.17	135	[65]
CI	Poly(methyl methacrylate)	In situ dispersion polymerization	4.5	151	[61]
Metallic cobalt	Polystyrene	Nitroxide-mediated polymerization	N.D.	38	[67]
Copper nickel alloy	Polyethylene glycol	Oil/water emulsion	N.D.	45	[68]
ZnFe_2_O_4_	Polyaniline	Pickering emulsion polymerization	5.4	73.7	[69]
